# Genomic selection of juvenile height across a single-generational gap in Douglas-fir

**DOI:** 10.1038/s41437-018-0172-0

**Published:** 2019-01-10

**Authors:** Frances R. Thistlethwaite, Blaise Ratcliffe, Jaroslav Klápště, Ilga Porth, Charles Chen, Michael U. Stoehr, Yousry A. El-Kassaby

**Affiliations:** 1 Department of Forest and Conservation Sciences, Faculty of Forestry, The University of British Columbia, 2424 Main Mall, Vancouver, BC V6T 1Z4 Canada; 2 Scion (New Zealand Forest Research Institute Ltd.), 49 Sala Street, Whakarewarewa, Rotorua, 3046 New Zealand; 3 Department of Genetics and Physiology of Forest Trees, Faculty of Forestry and Wood Sciences, Czech University of Life Sciences Prague, Praha 6, 165 21 Czech Republic; 4 Département des sciences du bois et de la forêt, Université Laval, G1V 0A6 Québec, QC Canada; 5 Department of Biochemistry and Molecular Biology, Oklahoma State University, Stillwater, OK 74078-3035 USA; 6 British Columbia Ministry of Forests, Lands and Natural Resource Operations, Victoria, BC V8W 9C2 Canada

**Keywords:** Plant breeding, Quantitative trait, Plant breeding, Quantitative trait

## Abstract

Here, we perform cross-generational GS analysis on coastal Douglas-fir (*Pseudotsuga menziesii*), reflecting trans-generational selective breeding application. A total of 1321 trees, representing 37 full-sib F_1_ families from 3 environments in British Columbia, Canada, were used as the training population for (1) EBVs (estimated breeding values) of juvenile height (HTJ) in the F_1_ generation predicting genomic EBVs of HTJ of 136 individuals in the F_2_ generation, (2) deregressed EBVs of F_1_ HTJ predicting deregressed genomic EBVs of F_2_ HTJ, (3) F_1_ mature height (HT35) predicting HTJ EBVs in F_2_, and (4) deregressed F_1_ HT35 predicting genomic deregressed HTJ EBVs in F_2_. Ridge regression best linear unbiased predictor (RR-BLUP), generalized ridge regression (GRR), and Bayes-B GS methods were used and compared to pedigree-based (ABLUP) predictions. GS accuracies for scenarios 1 (0.92, 0.91, and 0.91) and 3 (0.57, 0.56, and 0.58) were similar to their ABLUP counterparts (0.92 and 0.60, respectively) (using RR-BLUP, GRR, and Bayes-B). Results using deregressed values fell dramatically for both scenarios 2 and 4 which approached zero in many cases. Cross-generational GS validation of juvenile height in Douglas-fir produced predictive accuracies almost as high as that of ABLUP. Without capturing LD, GS cannot surpass the prediction of ABLUP. Here we tracked pedigree relatedness between training and validation sets. More markers or improved distribution of markers are required to capture LD in Douglas-fir. This is essential for accurate forward selection among siblings as markers that track pedigree are of little use for forward selection of individuals within controlled pollinated families.

## Introduction

There is a strong drive to incorporate genomic selection (GS) methodologies, as first proposed by Meuwissen et al. 2001, into forest tree selective breeding. With a proliferation of genomic technologies and a steady decline in genotyping costs (Heffner et al. 2010; Thomson 2014), breeders are taking full advantage of the availability of large SNP data sets. With these large SNP sets, it is envisaged that linkage disequilibrium (LD) between the markers and most sources of variation for valued complex phenotypes can be tracked. In doing so, capturing more variance than the well-established marker-assisted selection (MAS), which relies on fewer, large effect quantitative trait loci (QTLs) (El-Kassaby 1982). Genetically complex traits (such as height, growth, and wood quality) are now amenable to selection with the use of dense marker data. Given this statistical advantage, it is anticipated that GS may be implemented into tree selective breeding, as it has been done in livestock breeding (Van Eenennaam et al. 2014), resulting in higher genetic gain per unit time for traits of interest. This will largely be achieved through the reduction of trait evaluation time for such late expressing traits, leading to a faster turn-over in breeding generations, a significant time-sink in current breeding programs (Hayes et al. 2009; Heffner et al. 2010). Furthermore, breeding programs will become more dynamic as they will be able to ensure adaptation to capricious influences such as climate change and biotic disturbance in less time (Grattapaglia 2014).

Early deterministic simulations by Grattapaglia and Resende (2011) of GS that modeled forest tree species reported promising results. Following this, several experimental investigations have built upon this concept with varying success (Bartholomé et al. 2016; Beaulieu et al. 2014a, 2014b; Fuentes-Utrilla et al. 2017; Gamal El-Dien et al. 2015; Grattapaglia 2014; Isik et al. 2016; Müller et al. 2017a; Ratcliffe et al. 2015; Resende et al. 2012a, 2012b, 2012c, 2017; Tan et al. 2017; Thistlethwaite et al. 2017). In general, the following phases are involved in the GS process: (1) the genetic and phenotypic evaluation of a random subset of samples from within a selected population forming the training set from the tree breeding population under investigation; (2) creating a predictive model using this data, in which alleles at all marker loci have their effects simultaneously estimated; (3) implementing a validation or cross-validation process to test the developed models’ robustness; and (4) genomic prediction on a different subset of individuals from the same breeding population and selection of candidates from this population for next-generation breeding based on their genomic estimated breeding values (GEBVs) (Meuwissen et al. 2001; Grattapaglia 2014). The repercussion of this is a paradigm shift, in which the model unit of these breeding analyses shifts from being the line of descent to the allele.

Factors influencing the success of GS are varied, but one, which is entirely at the discretion of the investigator, is the statistical prediction method. Many methods have been proposed for GS and can be differentiated largely by their a priori assumptions of variance distribution. Ridge regression best linear unbiased prediction (RR-BLUP) and generalized ridge regression (GRR) have been selected for their computational efficiency. RR-BLUP assumes marker effects to be normally distributed (mean = 0) and to have equal variance. Conversely, GRR allows for heterogeneous variances and so employs a step that sets marker-specific shrinkage parameters on BLUP. In addition to these two methods, Bayes-B will also be implemented. The former have been shown to be highly sensitive to genetic relationships, leading to the accuracy of GEBV predictions based on markers tracking this relationship rather than LD (Habier et al. 2007; Thistlethwaite et al. 2017). This conclusion led Habier et al. (2007) to recommend the use of Bayes-B as an alternative.

Other features which independently and collectively alter the success of GS include: (1) The extent of LD between markers and QTL, (2) density of marker coverage; (3) training population size; (4) relatedness of samples (Habier et al. 2007), (5) trait heritability; (6) genetic architecture of trait (number of loci and effect size) (Hayes et al. 2009; Grattapaglia 2014); and (7) effective population size (*N*_*e*_) (Lorenz et al. 2011). Again features 2, 3, and 7 are (to a certain extent) under the control of the experimenter.

Regarding relatedness between individuals (feature 4), the parent average effect on EBVs was removed in this study following Garrick et al. (2009). The deregressed information is used as an alternative to EBVs in genomic selection to reduce the bias and increase reliability. Using the BLUP procedure shrinks both individual and progeny information toward the parent average EBV, and so there is motivation to remove this effect for the following rationale: (1) those records with an EBV but no individual or progeny information do not contribute to genomic estimation. Their EBV is calculated purely on the parental average and therefore do not afford any additional information besides that of the parental EBVs and genotypes and (2) to avoid the shrinking of major effects that are potentially segregating in the parents. Without deregression the EBVs of the offspring will all be shrunk toward the parent average, regardless of whether they inherit the favorable or unfavorable allele (Garrick et al. 2009).

The extent of marker-QTL LD is dictated by the relationship between *N*_*e*_ and the number of markers used (Grattapaglia and Resende 2011). In accordance with established theory, in a population with low *N*_*e*_, genetic drift has a stronger effect, resulting in an increase in non-random association of markers and QTL (LD). Thus, in this situation fewer markers are required to capture the variation of the trait of interest. In outbreeding populations, recombination negates LD and so, long-ranging LD is lost over generations. Because of this, models must be continually updated according to the distance between generations. Wu et al. (2015) found that increasing the generational gap between training and testing populations, reduced predictive accuracy of GS. Two factors can be garnered from this information, and their interaction is thought to have the highest impact on the success of GS in trees: (1) *N*_*e*_ must dictate the scale of marker density and (2) LD can be controlled through choosing the *N*_*e*_ (Grattapaglia 2014). Larger training population sizes were shown to increase predictive accuracy up to a threshold of around 1000 individuals, beyond which there were negligible gains (Grattapaglia and Resende 2011). Although Meuwissen et al. (2001) reported findings that suggested larger training populations negated somewhat the effects of low trait heritability, this had little effect on GS predictive accuracy of more complex traits (50–100 + QTL) (Grattapaglia and Resende 2011).

Substantial work has been carried out on testing the efficacy of GS in forest tree selective breeding. Validation populations (or cross-validation) have largely been comprised of individuals from within the same generation as the training population, aside from work performed by Isik et al. (2016) and Bartholomé et al. (2016). This practice has resulted in effectively side-stepping the issue of the generational gap. To address this issue, we performed a cross-generational GS analysis on coastal Douglas-fir (*Pseudotsuga menziesii* Mirb. (Franco)). The training population, on which the predictive models were trained, was collected from the parental generation (F_1_). The set was composed of 1321 randomly selected, 38-year-old trees, representing 37 full-sib families with replications over three environments in British Columbia (Canada) (Thistlethwaite et al. 2017). The validation population was collected from the progeny of the parental generation (F_2_, *n* = 136) with shared pedigree to the studied 37 full-sib families. A total of 69,551 SNPs were used in the GS analyses to produce a predictive model for juvenile height, which was compared to pedigree-based (ABLUP) predictions.

## Methods

### Experimental population

Samples from a 38-year-old replicated coastal Douglas-fir (*Pseudotsuga menziesii* Mirb. (Franco)) progeny testing population (F_1_) was used as the training set to develop the GS predictive models. The breeding program was established by the Ministry of Forests, Lands and Natural Resource Operations of British Columbia (BC), Canada in 1975. A total of 37 of 165 full-sib families were randomly selected for sampling from 3 environments in British Columbia, Canada (Adams (Lat. 50° 24′ 42″ N, Long. 126° 09′ 37″ W, Elev. 576 mas), Fleet River (Lat. 48° 39′ 25″ N, Long. 128° 05′ 05″ W, Elev. 561 mas), and Lost Creek (Lat. 49° 22′ 15″ N, Long. 122° 14′ 07″ W, Elev. 424 mas)). A total of 1321 individuals (*N*_*e*_ ≈ 21) from these 3 environments (Adams: *N* = 449, Fleet River: *N* = 441, Lost Creek: *N* = 431) were selected for genotyping and to train the GS models and ABLUP validation model. The *N*_*e*_ was estimated using a program developed by Dr. Milan Lstiburek (Faculty of Forestry and Wood Sciences, Czech University of Life Sciences Prague, Prague, Czech Republic) based on the status number concept of Lindgren et al. (1997).

The validation population (F_2_) is represented by 247 samples from control pollinated offspring derived from the 37 full-sib families described above with offspring from an additional 5 full-sib families selected from the same progeny testing population (F_1_) (42 F_2_ families total). ABLUP accuracies were derived using these 247 samples. Due to missing genotype information some F_2_ samples were discarded for GS analysis. The remaining 136 samples were used as the GS validation population, representing 17 parents located at Jordan River, BC (Lat. 48° 25′ 52.6N, Long. 124° 02′ 46.2W, Elev. 150 mas) and established in 2003.

In order to derive best possible estimates for the EBVs, an increased number of individuals (total *N* = 36,311) was used to provide as much information as possible when fitting the ABLUP model in ASReml 4.0 (Gilmour et al. 2009). Information was used from 11 environments of the 38-year-old progeny testing population (F_1_) (*N* = 33,931), plus their wild progenitors (*N* = 108) as described previously (Yanchuk 1996), an ungenotyped replicate of the F_2_ Jordan River validation population (*N* = 2025) (North Arm, Lat. 48° 50′ 41.7″ N, Long. 124° 06′ 34.8″ W), and the Jordan River environment itself (*N* = 247) were also incorporated into the analysis (total *N* = 36,311). The EBVs of a genotyped subset were used in the training of all models (Table 1). The “original” EBVs of the genotyped subset from Jordan River were used to validate each model.

**Table 1:** Summary of the number of individuals per environment, and to which models they contributed

Environment	*n*	Number genotyped	Generation	Model contribution	Heritability (*h*^2^)
Wild progenitors	108		P_0_	ABLUP^a^	NA
Adams	3478	449	F_1_	ABLUP, GS	0.19
Fleet River	2944	441	F_1_	ABLUP, GS	0.22
Lost Creek	3244	431	F_1_	ABLUP, GS	0.13
Sechelt	2909		F_1_	ABLUP	0.20
Squamish River	3153		F_1_	ABLUP	0.25
Eldred River	3395		F_1_	ABLUP	0.13
Tansky Creek	2974		F_1_	ABLUP	0.27
Sproat Lake	2881		F_1_	ABLUP	0.20
White River	3010		F_1_	ABLUP	0.17
Gold River	3067		F_1_	ABLUP	0.09
Menzies	2876		F_1_	ABLUP	0.17
Jordan River	247	136	F_2_	ABLUP, GS	0.39
North Arm	2025		F_2_	ABLUP	0.31

^a^Pedigree only, “GS” includes all three genomic selection methods (RR-BLUP, GRR, and Bayes-B), and only genotyped individuals were used in the construction and validation of these models. Heritability was calculated within environments (with *n* individuals), using a full pedigree containing all generations

### Phenotyping, deregression, tissue sampling, DNA extraction, and genotyping

Early-rotation (juvenile height) (1988 for the training population and 2010 for the validation population) height measurements of the studied trees were recorded (HTJ: in cm). EBVs for HTJ were obtained in ASReml 4.0 (Gilmour et al. 2009) and used as phenotypes for the genomic prediction analysis. In addition, the EBVs were deregressed and parental averages removed, using the method “Removing parent average effects” proposed by Garrick et al. (2009). The resulting deregressed estimated breeding values (DEBVs) were used as alternative phenotypes for GS analysis. This type of deregressed data can be obtained by approximating and back-solving the evaluation equations. The following equations were solved for each individual tree:

1 $$\left[ {\begin{array}{*{20}{c}} {{\mathbf{Z}}\prime _{{\mathrm{PA}}}{\mathbf{Z}}_{{\mathrm{PA}}} + 4\lambda } & { - 2\lambda } \\ { - 2\lambda } & {{\mathbf{Z}}\prime _{i - {\mathrm{PA}}}{\mathbf{Z}}_{i - {\mathrm{PA}}} + 2\lambda } \end{array}} \right]\left[ {\begin{array}{*{20}{c}} {{\mathbf{PA}}} \\ {{\mathbf{EBV}}} \end{array}} \right] = \left[ {\begin{array}{*{20}{c}} {y_{{\mathrm{PA}}}} \\ {y_{i - {\mathrm{PA}}}} \end{array}} \right]{,}$$

where **PA** and **EBV** represent the parental average and estimated breeding value vectors, respectively; $y_{{\mathrm{PA}}}$ and $y_{i - {\mathrm{PA}}}$ represent information equivalent to the right-hand-side elements referring to the PA and individual respectively; *λ* = (1 − *h*^*2*^)/*h*^*2*^; ${\mathbf{Z}}\prime _{{\mathrm{PA}}}{\mathbf{Z}}_{{\mathrm{PA}}}$ and ${\mathbf{Z}}\prime _{i - {\mathrm{PA}}}{\mathbf{Z}}_{i - {\mathrm{PA}}}$ express the unknown information content of the parental average, and individual effect without parental average, respectively. These latter terms can be equated by solving firstly Eq. (2) and using the result to solve for Eq. (3):

2 $${\mathbf{Z}}\prime _{{\mathrm{PA}}}{\mathbf{Z}}_{{\mathrm{PA}}} = \lambda \left( {0.5\alpha - 4} \right) + 0.5\lambda \sqrt {\left( {\alpha ^2 + \frac{{16}}{\delta }} \right)}{,}$$

3 $${\mathbf{Z}}\prime _{i - {\mathrm{PA}}}{\mathbf{Z}}_{i - {\mathrm{PA}}} = \delta {\mathbf{Z}}\prime _{{\mathrm{PA}}}{\mathbf{Z}}_{{\mathrm{PA}}} + 2\lambda (2\delta - 1){,}$$

where *α* = 1/(0.5 *−* $r_{{\mathrm{PA}}}^2$); and *δ* = (0.5 − $r_{{\mathrm{PA}}}^2$)/(1 − $r_i^2$). $r_{{\mathrm{PA}}}^2$ is defined as the reliability of the PA for individual *i* with parents “*sire*” and “*dam*”, and can be calculated by: $r_{{\mathrm{PA}}}^2 = \frac{{r_{sire}^2 + r_{dam}^2}}{4}$. While $r_i^2$ the reliability of the EBV, was calculated as the square of the correlation between the true and predicted breeding values (*r*_*i*_) according to Gilmour et al. (2009):

4 $$r_i = \sqrt {1 - \frac{{s_i^2}}{{(1 + f_i)\sigma _A^2}}}$$

where $s_i^2$ is the prediction error variance for individual *i*; *f*_*i*_ is the inbreeding coefficient for individual *i* calculated in ASReml 4.0 (Gilmour et al. 2009); and $\sigma _A^2$ is the additive genetic variance.

We can now complete and solve the coefficient matrix (Eq. (1)) using Eqs. (2) and (3), and multiply this by the vectors PA and EBV. The deregressed information regarding the individual without PA effects is obtained using this simplified formula:

5 $$\frac{{y_{i - {\mathrm{PA}}}}}{{{\mathbf{Z}}\prime _{i - {\mathrm{PA}}}{\mathbf{Z}}_{i - {\mathrm{PA}}}}}$$

Cambial tissue was collected from the mature trees of the training population; this was an elegant solution to overcome the difficulty of obtaining foliage tissue from older/taller trees. Using a hammer and punch tool (approx. 2 cm diameter) two small circular disks of bark, cambium and developing tissue were removed from each tree. Once separated, the cambial tissue was immediately stored in a 2 ml collection tube with 1 ml of storage buffer (10 mM EDTA pH 8.0, 10 mM Na_2_SO_3_), these were kept at 4 °C until DNA extraction. Foliage DNA extraction is easier using a standard protocol, therefore leaf bud tissue was collected from the juvenile trees. Two samples from each juvenile tree were taken and stored in the same way as the cambial tissue (in 1 ml of storage buffer and kept at 4 °C). The same DNA extraction protocol was used on both forms of the tissue samples. This was a modified protocol developed by Ivanova et al. 2008 (R. Whetten, unpublished, North Carolina State University, personal communications). Whole exome capture genotyping was carried out in a commercial facility (RAPiD Genomics^©^, FL, USA), with probes designed using the available Douglas-fir transcriptome assembly (Howe et al. 2013). For further details on the genotyping process see Thistlethwaite et al. (2017), both the training and validation populations were genotyped at the same time using the same procedure. For more information regarding exome capture, see Neves et al. (2013).

### EBV prediction and accuracy

ASReml 4.0 (Gilmour et al. 2009) was used to fit EBVs, using information from the 11 F_1_ parental environments, their parents (P_0_) and the 2 F_2_ juvenile environments (total *N* = 36,311) (Table 1). As environmental effects are an important consideration in forestry (Cappa et al. 2016), and to account for site (environment) and age differences, a linear mixed model analysis was carried out,

6 $$y = {\boldsymbol{X}}\beta + {\boldsymbol{Z}}_1a + {\boldsymbol{Z}}_2sa + {\boldsymbol{Z}}_3s(rep) + {\boldsymbol{Z}}_4sf + {\boldsymbol{Z}}_5f + e{,}$$

where *y* is the phenotypic trait measurement; *β* is a vector of fixed effects (i.e., mean, site, and age effects); *a* is a vector of individual random additive effects following ~*N*(0, ***A***σ_a_^2^); *sa* is a site × additive genetic interaction following ~*N*(0, ***I***σ_sa_^2^); *s*(*rep*) is a vector of the block effect nested within site following ~*N*(0, ***I***σ_s(rep)_^2^); *sf* is a random effect site × family interaction following ~*N*(0, ***I***σ_sf_^2^); *f* is the effect of family and following ~*N*(0, ***I***σ_f_^2^); and *e* is the random residual effect following ~*N*(0, ***I***σ_e_^2^); ***X*** and ***Z***_***1-5***_ are incidence matrices assigning fixed and random effects to each observation at vector *y*; lastly ***I*** is the identity matrix and ***A*** the average numerator relationship matrix (Wright 1922). We chose to use a common variance for all environments since this is the most parsimonious model when using a large number of environments. This avoids over-fitting the model since the number of parameters increases much faster than the number of environments (Isik et al. 2017). Theoretical accuracy of the EBVs ($\hat r$) was calculated following Dutkowski et al. (2002).

7 $$\hat r = \sqrt {1 - \frac{{SE_i^2}}{{(1 + F_i)\hat \sigma _a^2}}}{,}$$

where *SE*_*i*_ is the standard error of breeding value, and *F*_*i*_ is the inbreeding coefficient of the *i*th individual. Narrow-sense heritability was calculated as *h*^2^ = *σ*_a_^2^/(*σ*_a_^2^ + *σ*_sa_^2^ + *σ*_sf_^2^ + *σ*_f_^2^ + *σ*_e_^2^), where *σ*_a_^2^, *σ*_sa_^2^, *σ*_sf_^2^, *σ*_f_^2^, and *σ*_e_^2^ are the variances of additive genetic, site × additive genetic, site × family, family, and residual effects, respectively.

ABLUP validation was carried out in ASReml R v4.1, predicting the breeding values of the validation population using an expected relationship matrix (***A***) based on pedigree information. A tenfold validation approach was used. Briefly, samples from the F_1_ parental generation at Adams, Fleet River, and Lost Creek (*N* = 1321) were randomly partitioned into ten training subsets. Nine of these subsets (approximately 90% of the F_1_ samples) were used as the training set, on which the ABLUP model would be trained to estimate breeding values. On the basis of this model training, EBVs of the validation set were predicted. The validation set was composed of the 136 individuals from the F_2_ Jordan River environment. This was repeated ten times until all F_1_ subsets had been included in the training set. Then the whole process was repeated ten times, randomly assigning the training subsets. Prediction accuracy was measured as the correlation between the predicted EBVs from the cross-validation, and their original EBVs calculated using all possible pedigree information. In addition, the predictive ability was calculated as the correlation between predicted EBVs and actual height phenotypes.

### Genomic selection analysis

Three statistical methods were used to perform genomic selection: RR-BLUP, GRR, and Bayes-B (Lorenz et al. 2011). Four GS analyses were performed: (1) models were trained on EBVs for juvenile height of the F_1_ trees, GEBVs for height of the F_2_ validation set were predicted (HTJ EBVs → HTJ GEBVs); (2) models were trained on DEBVs for juvenile height of the F_1_ trees, genomic estimates of deregressed breeding values (GDEBVs) for height of the F_2_ validation set were predicted (HTJ DEBVS → HTJ GDEBVs); (3) GS models were trained on EBVs of mature height (age 35) of the F_1_ samples and GEBVs of the F_2_ validation set were predicted and correlated with their juvenile EBVs to ascertain any relationship (HT35 EBVs → HTJ GEBVs); (4) models were trained on DEBVs for mature height of the F_1_ samples, GDEBVs of the F_2_ validation set were predicted and correlated with their juvenile DEBVs (HT35 DEBVs → HTJ GDEBVs). A tenfold validation process repeated ten times, was again used to randomly select individuals from the F_1_ generation to construct the training set of the models. Prediction accuracy for each model in all four analyses was calculated as the mean of the replications of the Pearson product-moment correlation between the original EBVs (as calculated with all pedigree information *N* = 36,311) for HTJ of the 136 F_2_ validation trees from Jordan River and their predicted GEBVs. Alternatively, using DEBVs to train the models, the prediction accuracy is the Pearson product-moment correlation between DEBVs of the validation set and their predicted GDEBVs. Similarly to the ABLUP investigation, the predictive ability was calculated as the correlation between predicted GEBVs and actual height phenotypes.

### Ridge regression best linear unbiased predictor

RR-BLUP (Whittaker et al. 2000) was implemented using the R package “bigRR” (Shen et al. 2014). The predicted heights (or GEBVs) are obtained by the summing of all the marker effects of an individual tree. Marker effects were estimated as in Henderson (1976), under the following mixed model:

8 $${\boldsymbol{y}}_{{\boldsymbol{D}}({\boldsymbol{EBV}})} = \vec 1{\boldsymbol{\mu }} + {\boldsymbol{Zg}} + {\boldsymbol{e}}{,}$$

where ${\boldsymbol{y}}_{{\boldsymbol{D}}({\boldsymbol{EBV}})}$ is the vector of *n* tree height records (EBVs or DEBVs in this case), $\vec 1$ is a vector of 1, ***μ*** is an intercept, ***g*** is the vector of random marker effects, ***Z*** is the design matrix for the random marker effects, and ***e*** is the residual vector for random effects. In RR-BLUP the residuals and marker effects follow normal distributions with constant variance, i.e., *e* ~ *N* (0, ***I***$\sigma _e^2$) and *g* ~ N(0, ***I***$\sigma _g^2$), where ***I*** is an identity matrix. The solution for the marker effects is given by the following equation:

9 $${\hat{\boldsymbol {g}}} = \left( {{\boldsymbol{Z}}\prime {\boldsymbol{Z}} +{\lambda} {\boldsymbol{I}}} \right)^{ - 1}{\boldsymbol{Z}}\prime {\boldsymbol{y}}$$

where *ʎ* = $\sigma _e^2/\sigma _g^2$ is the ridge penalization parameter. An assumption of this method is that all marker effects are distributed equally, and therefore all effects are equally shrunk towards zero.

### Generalized ridge regression

GRR was implemented in the R package “bigRR” (Shen et al. 2014). The first step, in this two-step variable selection method, is to use linear mixed models optimizing *ʎ*, to estimate marker effects (the same as RR-BLUP). Where it differs from RR-BLUP is in a second step. In which an alternative, marker-specific shrinkage parameter is imposed on the BLUP for ${\hat{\boldsymbol g}}.$

In this heterogeneous error model, *ʎ****I*** becomes *diag*(*ʎ*) in Eq. (4):

10 $${\hat{\boldsymbol g}} = ({\boldsymbol{Z}}\prime {\boldsymbol{Z}} + diag(\lambda))^{ - 1}{\boldsymbol{Z}}\prime {\boldsymbol{y}}$$

Here *ʎ* is a vector of *p* shrinkage parameters. For the *k*th element: *ʎ*$_k = \widehat {\sigma _e^2}/\widehat {\sigma _{gk}^2}$, is the parameter, where $\widehat {\sigma _{gk}^2}$ is the variance of marker effect *k* ($\widehat {\sigma _{gk}^2} = \hat g_k^2/(1 - h_{kk})$). Where $\hat g$ is the BLUP marker effect (from step 1), and $h_{kk}$ is the effect of the dependant variable on the fitted value for observation *k*. To wit, $h_{kk}$ represents the diagonal element (*n* + *k*) of the influence matrix ***H*** = ***T(T’T)***^−*1*^***T’***, and

11 $$T = \left( {\begin{array}{*{20}{c}} {\vec 1} & Z \\ 0 & {diag(\lambda)} \end{array}} \right)$$

### Bayes-B

Bayesian methods, as first proposed by Meuwissen et al. (2001), seek to relax the assumption that genetic effects are evenly distributed across the genome (as in RR-BLUP). In this analysis, we use Bayes-B, another variable selection method, in which there exists a probability that a marker has no effect (π). This would correspond to a situation where the genetic architecture of the trait was such that genetic variance was present at few, major effect loci only (Heffner et al. 2009; Lorenz et al. 2011). Bayes-B is thought to be a more realistic prior since some genomic regions will be absent of QTL (Heffner et al. 2009). An assumption of this model is that marker effects are normally distributed with zero mean and finite variance. The prior distribution of the marker effect variance, is a mixture of two finite prior densities: var (*g*) = 0, with probability π; and var (*g*) ~ *χ*^−2^ (v, S), with probability (1 − π) (Lorenz et al. 2011; Gezan et al. 2017). π is assumed known and specified arbitrarily, the default value of 0.5 was used. The Bayes-B analysis was carried out in the R package BGLR v1.0.4 (Perez and de los Campos 2014), with the Gibbs sampler run for 100,000 iterations and a burn-in of 20,000, with a thinning rate of 100. Default rules of the BGLR R package (Perez and de los Campos 2014) were used for the initial hyper-parameter values.

The data sets supporting the results of this article will be available in the Dryad Digital Repository upon acceptance.

## Results

### Heritability and EBV accuracy

Juvenile height (HTJ) heritability was estimated using a pedigree-based relationship matrix (ABLUP), including individuals from the 11 parental (F_1_) environments, their parents (P_0_), and the 2 progeny (F_2_) environments. A summary of the contribution of each environment to both the ABLUP and GS models is in Table 1, along with environment heritabilities for HTJ which ranged from 0.09 to 0.39. The overall HTJ heritability estimate was 0.14 (SE 0.025). The average theoretical accuracy for the EBVs of the sampled, genotyped individuals (from 3 parental environments: Adams, Fleet River and Lost Creek; and 1 progeny environment: Jordan River) was 0.68, and 0.61 for the validation environment (Jordan River) alone.

### Validation in the progeny generation

#### ABLUP

The average prediction accuracy for Jordan River EBVs derived from ABLUP was 0.92 (SE 0.001) (Table 2), using a pedigree including genotyped samples only, i.e., the validation set and the F_1_ samples from the three environments (Adams, Fleet River, and Lost Creek). Using a larger pedigree based on the full 11 F_1_ environments (plus their parents), and two progeny environments, the prediction accuracy becomes 0.95 (SE 0.0005). While the pedigree for the ABLUP analyses included multiple generations, phenotypic information from the F_1_ generation only was used to predict the validation (F_2_) EBVs in the cross-validation. For comparison with the GS analyses, it is these results that we shall concentrate on. The average predictive ability for HTJ using ABLUP was calculated as the correlation between EBV and juvenile height measurements. The predictive ability of ABLUP in the validation set was calculated as 0.71 (SE 0.003) using a pedigree including genotyped samples only, i.e., the validation set from Jordan River (F_2_) and the F_1_ samples from the three environments: Adams, Fleet River, and Lost Creek (Table 3). Higher than the theoretical accuracy (0.61) calculated for the validation environment (Jordan River) alone.

**Table 2:** Genomic selection analyses of four models using three GS statistical methods (RR-BLUP, GRR, and Bayes-B)

Analysis	Accuracy (SE)			
	ABLUP	RR-BLUP	GRR	Bayes-B
**HTJ EBVs** **→** **HTJ GEBVs**	**0.92 (0.001)**	**0.92 (0.0002)**	**0.91 (0.0003)**	**0.91 (0.0007)**
EBV < 20^1^		0.43 (0.003)	0.42 (0.003)	0.42 (0.004)
EBV < 20^2^		0.43 (0.004)	0.38 (0.005)	0.38 (0.005)
EBV < 20^3^		0.45 (0.006)	0.43 (0.006)	0.44 (0.005)
EBV > 20^1^		−0.005 (0.004)	−0.05 (0.004)	−0.02 (0.006)
EBV > 20^2^		−0.04 (0.004)	−0.11 (0.004)	−0.10 (0.005)
EBV > 20^3^		0.54 (0.009)	0.52 (0.010)	0.99 (0.0001)
**HTJ DEBVS** **→** **HTJ GDEBVs**		**0.10 (0.008)**	**0.05 (0.007)**	**0.48 (0.002)**
EBV < 20^1^		−0.12 (0.004)	−0.11 (0.004)	−0.02 (0.005)
EBV < 20^2^		−0.12 (0.004)	−0.14 (0.004)	−0.09 (0.005)
EBV < 20^3^		−0.08 (0.004)	−0.07 (0.004)	−0.07 (0.004)
EBV > 20^1^		0.17 (0.004)	0.26 (0.004)	0.16 (0.006)
EBV > 20^2^		0.14 (0.004)	0.17 (0.004)	0.13 (0.006)
EBV > 20^3^		−0.23 (0.003)	−0.22 (0.003)	0.09 (0.009)
**HT35 EBVs** **→** **HTJ GEBVs**	**0.60 (0.010)**	**0.57 (0.002)**	**0.56 (0.002)**	**0.58 (0.003)**
EBV < 20^1^		0.15 (0.004)	0.20 (0.004)	0.04 (0.007)
EBV < 20^2^		0.13 (0.004)	0.18 (0.004)	0.04 (0.007)
EBV < 20^3^		0.35 (0.005)	0.41 (0.005)	0.36 (0.007)
EBV > 20^1^		−0.21 (0.002)	−0.24 (0.002)	−0.28 (0.004)
EBV > 20^2^		−0.22 (0.002)	−0.25 (0.002)	−0.30 (0.003)
EBV > 20^3^		−0.23 (0.003)	−0.22 (0.003)	−0.27 (0.003)
**HT35 DEBVs** **→** **HTJ GDEBVs**		**−0.15 (0.005)**	**−0.11 (0.005)**	**0.11 (0.007)**
EBV < 20^1^		−0.08 (0.003)	−0.02 (0.003)	−0.02 (0.003)
EBV < 20^2^		−0.08 (0.003)	−0.07 (0.003)	−0.06 (0.003)
EBV < 20^3^		0.06 (0.002)	0.07 (0.003)	0.06 (0.003)
EBV > 20^1^		0.02 (0.004)	0.07 (0.004)	−0.06 (0.004)
EBV > 20^2^		0.02 (0.005)	0.03 (0.006)	−0.07 (0.003)
EBV > 20^3^		0.07 (0.004)	0.08 (0.004)	−0.01 (0.006)

ABLUP is a pedigree only model with no marker information used. Results are from the validation procedure replicated 10 times, in which a random 90% of the genotyped F_1_ generation (1321 trees from Adams, Fleet River, and Lost Creek) was used as the training set and the validation set was comprised of the 136 genotyped F_2_ trees from Jordan River. Accuracy was calculated as the mean of the replications of the Pearson product-moment correlation between the original EBVs for HTJ of the 136 F_2_ validation trees from Jordan River and their predicted GEBVs or GDEBVs. The four analyses are: F_1_ juvenile height EBVs predicting F_2_ juvenile height EBVs (HTJ EBVs → HTJ GEBVs); F_1_ juvenile height DEBVs predicting F_2_ juvenile height DEBVs (HTJ DEBVS → HTJ GDEBVs); F_1_ mature (age 35) height EBVs predicting F_2_ juvenile height EBV (HT35 EBVs → HTJ GEBVs); and F_1_ mature height DEBVs predicting F_2_ juvenile height GDEBVs (HT35 DEBVs → HTJ GDEBVs). Results for the validation set as a whole are in bold (*N* = 136), following these are the results for each of the two clusters EBV < 20 (*N* = 83) and EBV > 20 (*N* = 53), with indices representing different training set composition: ^1^ all genotyped F_1_ individuals; ^2^ all genotyped F_1_ individuals minus the parents of the opposing cluster; ^3^ only the F_1_ parents of the cluster in question

**Table 3:** The corresponding predictive abilities for GS analyses in Table 2, calculated as the correlation between the raw phenotype (juvenile height: HTJ) and their genomic estimated breeding values (GEBVs) or deregressed genomic estimated breeding values (GDEBVs)

Analysis	Predictive ability (SE)			
	ABLUP	RR-BLUP	GRR	Bayes-B
**r (HTJ, HTJ GEBVs)**	**0.71 (0.003)**	**0.43 (0.0004)**	**0.42 (0.0006)**	**0.43 (0.0007)**
EBV < 20^1^		0.10 (0.002)	0.07 (0.002)	0.07 (0.003)
EBV < 20^2^		0.04 (0.002)	−0.01 (0.003)	−0.01 (0.003)
EBV < 20^3^		−0.10 (0.003)	−0.12 (0.004)	−0.09 (0.003)
EBV > 20^1^		0.05 (0.002)	0.03 (0.002)	0.07 (0.004)
EBV > 20^2^		0.04 (0.002)	0.01 (0.002)	0.07 (0.003)
EBV > 20^3^		0.15 (0.004)	0.12 (0.005)	0.59 (0.0004)
**r (HTJ, HTJ GDEBVs)**		**0.06 (0.008)**	**0.009 (0.007)**	**0.40 (0.003)**
EBV < 20^1^		−0.20 (0.006)	−0.21 (0.005)	−0.04 (0.006)
EBV < 20^2^		−0.22 (0.005)	−0.25 (0.005)	−0.15 (0.007)
EBV < 20^3^		−0.21 (0.006)	−0.20 (0.006)	−0.21 (0.006)
EBV > 20^1^		0.23 (0.004)	0.31 (0.005)	0.20 (0.005)
EBV > 20^2^		0.19 (0.005)	0.22 (0.005)	0.16 (0.006)
EBV > 20^3^		0.15 (0.007)	0.11 (0.007)	0.07 (0.010)
**r (HTJ, HT35 GEBVs)**	**0.24 (0.005)**	**0.21 (0.001)**	**0.21 (0.001)**	**0.22 (0.002)**
EBV < 20^1^		−0.19 (0.001)	−0.13 (0.002)	−0.09 (0.005)
EBV < 20^2^		−0.23 (0.001)	−0.17 (0.002)	−0.14 (0.004)
EBV < 20^3^		−0.10 (0.002)	−0.07 (0.002)	−0.06 (0.004)
EBV > 20^1^		−0.03 (0.001)	−0.04 (0.001)	−0.09 (0.002)
EBV > 20^2^		−0.03 (0.0009)	−0.05 (0.001)	−0.07 (0.002)
EBV > 20^3^		−0.04 (0.001)	−0.04 (0.002)	−0.06 (0.001)
**r (HTJ, HT35 GDEBVs)**		**−0.17 (0.005)**	**−0.13 (0.004)**	**0.04 (0.006)**
EBV < 20^1^		−0.18 (0.003)	−0.13 (0.003)	−0.13 (0.003)
EBV < 20^2^		−0.18 (0.003)	−0.18 (0.003)	−0.18 (0.003)
EBV < 20^3^		−0.05 (0.003)	−0.03 (0.004)	−0.05 (0.004)
EBV > 20^1^		0.08 (0.005)	0.13 (0.004)	0.003 (0.004)
EBV > 20^2^		0.09 (0.005)	0.09 (0.006)	−0.01 (0.003)
EBV > 20^3^		0.15 (0.005)	0.15 (0.005)	0.06 (0.006)

Results for the validation set as a whole are in bold (*N* = 136), below each are the results for each of the two clusters EBV < 20 (*N* = 83) and EBV > 20 (*N* = 53), with indices representing different training set composition: 1 all genotyped F1 individuals; 2 all genotyped F1 individuals minus the parents of the opposing cluster; 3 only the F1 parents of the cluster in question

#### GS Scenario 1: HTJ EBVs → HTJ GEBVs

**Fig. 1:**
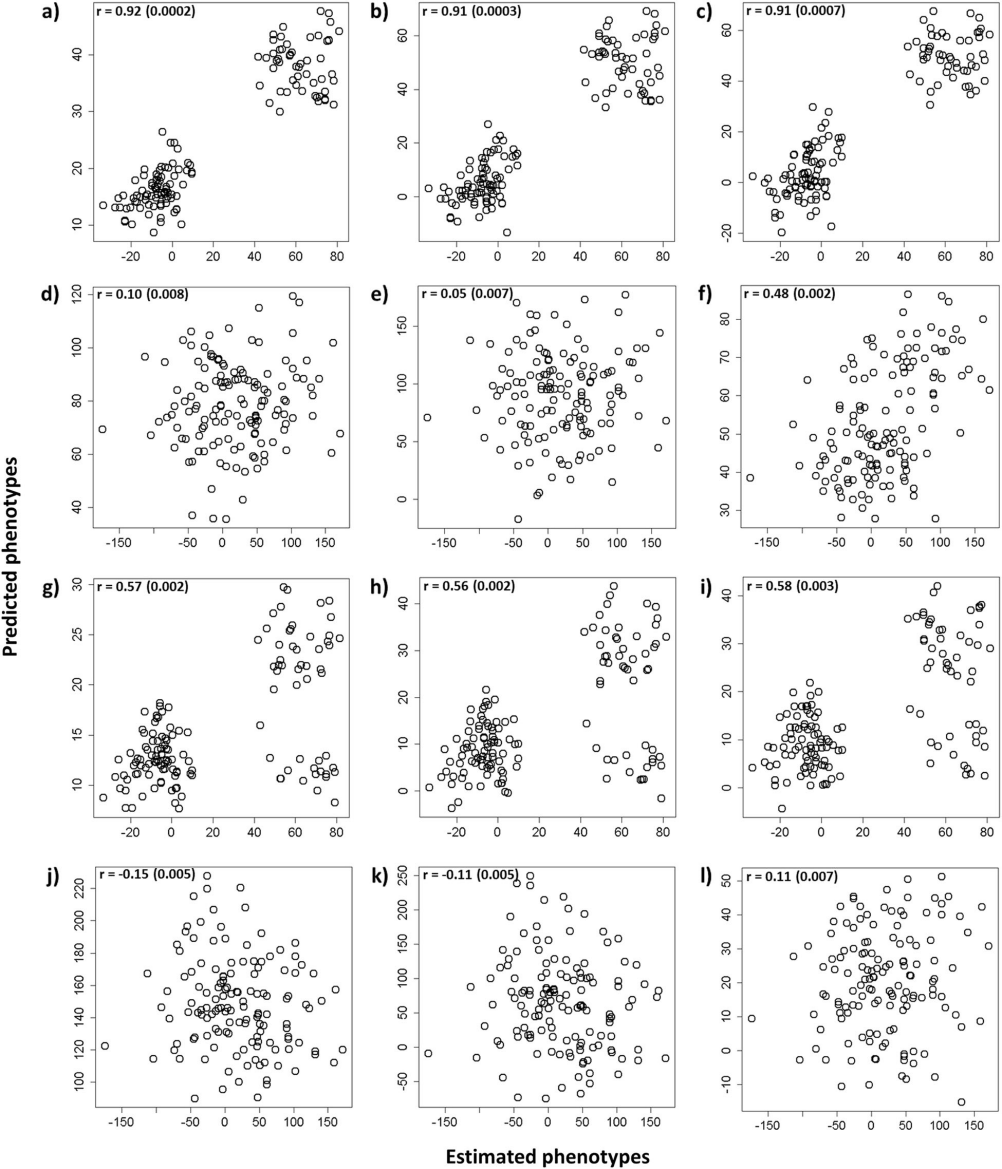
GS correlation in the validation set (correlation in the validation set of: EBVs and genomic estimated breeding values (GEBVs) for juvenile height (HTJ) using **a** RR-BLUP, **b** GRR, and **c** Bayes-B; deregressed estimated breeding values (DEBVs) and genomic deregressed estimated breeding values (GDEBVs) using **d** RR-BLUP, **e** GRR, and **f** Bayes-B; EBVs for HTJ vs. GEBVs for mature height age 35 years (HT35) using **g** RR-BLUP, **h** GRR, and **i** Bayes-B; and DEBVs for HTJ vs. DGEBVs for HT35 using **j** RR-BLUP, **k** GRR, and **l** Bayes-B)

Prediction accuracies for GEBVs derived from RR-BLUP, GRR, and Bayes-B are shown in Fig. 1. In the first cross-generational GS analysis, GS prediction accuracies for validation were determined by the correlation between EBV and GEBVs for the F_2_ validation (progeny generation) set at Jordan River. GS prediction accuracies with models trained on F_1_ EBVs were very similar over all GS methods used (Table 2), the average was 0.91. Their corresponding predictive abilities were also similar to each other with an average of 0.43, somewhat lower than that of ABLUP despite similar prediction accuracies (Table 3).

As is evident from the Fig. 1a–c, there is a pattern of distinct grouping characterized by those individuals with EBV over 20 and those below. We suspected this was the major factor in causing such high prediction accuracies. To this end, we further analyzed these groups, or clusters, separately, each with 3 different training set combinations represented by indicies 1-3. EBV<20^1^, EBV < 20^2^, and EBV < 20^3^ represent the analysis of validation individuals within the lower cluster, using: ^1^all genotyped F_1_ individuals (1321 trees from Adams, Fleet River, and Lost Creek) as the training set; ^2^ all genotyped F_1_ individuals minus the parents of the high cluster as the training set (total = 1104); and ^3^using only their parents from the F_1_ generation as the training set (total = 132). Similarly EBV > 20^1^ represents the analysis of validation individuals within the higher cluster (EBV > 20), using all genotyped F_1_ individuals as the training set (total = 1321); EBV > 20^2^ is the analysis of validation individuals within the high cluster, using all genotyped F_1_ individuals minus the parents of the low cluster as the training set (total = 1189); and EBV > 20^3^ is the analysis of validation individuals within the high cluster, using only their parents from the F_1_ generation as the training set (total = 217).

We subsequently found more limited correlations between EBVs and GEBVs within these clusters. When analyzed separately the lower cluster, with individuals with EBVs lower than 20 (hereafter referred to as EBV < 20), observed similar yet moderate predictive accuracies across all training set combinations and GS statistical methods (Table 2), obtaining an average of 0.42. GS method RR-BLUP and training set EBV < 20^3^ performed only slightly better than the other combinations. The higher cluster, the group with individuals with EBV over 20 (hereafter referred to as EBV > 20), observed markedly low prediction accuracies for EBV > 20^1^ and EBV > 20^2^ (average: −0.05). Yet with a training set comprised only of the parents of this cluster (EBV > 20^3^), that rose to an average of 0.68 over all GS methods (Table 2). Bayes-B, in this case, outperformed the other GS methods significantly, and indeed provided the best prediction accuracy in this investigation (0.99), greater than that of the ABLUP model.

#### GS Scenario 2: HTJ DEBVs → HTJ GDEBVs

The second GS analysis used the correlation between DEBVs and GDEBVs, of the F_2_ validation set at Jordan River, as an indication of predictive accuracy. Deregression was carried out using the procedure of Garrick et al. (2009), to account for family means and their effect on EBVs. The resulting deregressed EBVs (DEBVs) contain information regarding individuals only, without influence from parental BVs. Model accuracy fell dramatically when trained on DEBVs of the F_1_ training set rather than on EBVs, 0.10 (SE 0.008) for RR-BLUP (Fig. 1d), 0.05 (SE 0.007) for GRR (Fig. 1e), and 0.48 (SE 0.002) for Bayes-B (Fig. 1f). The Bayes-B analysis being noticeably higher than the other two analyses due to the aforementioned clustering structure of the validation set. Similarly, the predictive abilities of these three models were much lower for RR-BLUP and GRR and moderate for Bayes-B: 0.06 (SE 0.008), 0.009 (SE 0.007), and 0.40 (SE 0.003), respectively (Table 3). In this scenario, Bayes-B seemed to out-perform the other two GS models, however, the appearance of clustering in Fig. 1f called for further investigation.

Again, separate analyses were carried out according to the grouping of individuals mentioned previously. Although it is not immediately obvious in Fig. 1d, e, there is the emergence of a grouping pattern in Fig. 1f. The average predictive accuracy for EBV < 20 across all training set combinations and GS methods was −0.09 and the average predictive ability for EBV < 20 was −0.19. For EBV > 20 the average predictive accuracy of the training set combinations EBV > 20^1^ and EBV > 20^2^ was 0.17. This contrasts the results using only the parents of this high cluster as the training set, which gives an average predictive accuracy of −0.12 (Table 2). The average predictive ability for EBV > 20 was 0.18 (Table 3).

#### GS Scenario 3: HT35 EBVs → HTJ GEBVs

The third GS analysis, using EBVs for mature height (age 35) of the F_1_ samples as the training set for the models, and juvenile height GEBVs of the F_2_ generation at Jordan River as the validation set. The prediction accuracies achieved were similar between all three GS models, with an average of 0.57 (Fig. 1g–i). They were comparable but lower than the result of the ABLUP analysis in which HT35 was used to predict HTJ EBVs (0.60, SE 0.010) (Table 2). Likewise, their predictive abilities were generally quite similar to that of the ABLUP analysis (0.24, SE 0.005) with an average of 0.21 across all GS methods (Table 3):

The appearance of groups again appears in Fig. 1g–i as a result of individual EBV in the validation set. For the group EBV < 20 the prediction accuracy was on average 0.12 for EBV < 20^1^ and EBV < 20^2^. However, this increased to an average of 0.37 using a training set made up of the parents of the low cluster only. Prediction accuracy for the EBV > 20 cluster was more consistent across all training sets and GS methods (Table 2), with an average of −0.25. Although in this latter case the predictive accuracy correlation is driven by the presence of two smaller sub-groups within the EBV > 20 group. These subgroups have limited structure on their own.

#### GS Scenario 4: HT35 DEBVs → HTJ GDEBVs

Finally in the fourth analysis, using DEBVs for mature height of the F_1_ samples as the training set for the models, and GDEBVs for juvenile height of the F_2_ generation at Jordan River as the validation set; the average prediction accuracy or correlation between DEBVs and GDEBVs was −0.05 across all GS methods (Fig. 1j–l). The average predictive ability for this scenario was −0.09. All results were much lower than those for the ABLUP analysis for HT35 predicting HTJ EBVs (Tables 2 and 3).

The same groupings as before were tested for their prediction accuracies within the fourth analysis. The predictive accuracies for EBV < 20 were generally very low across all training set combinations and GS methods. The average for EBV < 20^1^ and EBV < 20^2^ was −0.06. With the direction of the linear relationship changing when only the EBV < 20 parents made up the training set (Table 2). The average for this training set combination over all GS methods was 0.06. Their average predictive abilities were: −0.16 for EBV < 20^1^ and EBV < 20^2^; with a slight drop in predictive ability in EBV < 20^3^ to −0.04. For EBV > 20 the predictive accuracies were similar in magnitude for all training set combinations and GS methods, with an average of 0.02. However, whilst RR-BLUP and GRR gave slight positive correlations, Bayes-B results were all negative in their direction. All within-cluster results were close to zero for this scenario, although the variation in the sign of the linear relationship causes some doubt as to their reliability. The predictive abilities for EBV > 20 were on average 0.08, predictably with the limited training set of EBV > 20^3^ having slightly higher predictive abilities than the other training sets (for RR-BLUP and GRR only).

## Discussion

### Heritability of juvenile height

The estimated heritability of juvenile height was substantially low (0.14), but only slightly lower than previous studies suggest for Douglas-fir given the age of the trees used in the estimation (Yeh and Heaman 1982; Dean and Stonecypher 2006; Ukrainetz et al. 2008; Thistlethwaite et al. 2017). However, it should be noted that information from many more environments was used to estimate this heritability (*N* = 36,311), increasing the genotype × environmental interaction, effectively shrinking the heritability. Using only the 1321 F_1_ trees, the heritability estimate is 0.17. The effect of heritability appears to be minimal in this case, since high predictive accuracies were obtained for validation models for HTJ predicting HTJ: 0.92 for ABLUP, 0.92, 0.91, and 0.91 for RR-BLUP, GRR, and Bayes-B, respectively (Fig. 1a–c), using EBVs as the GS model input; and for HT35 predicting HTJ: 0.60 for ABLUP, 0.57, 0.56, and 0.58 for RR-BLUP, GRR, and Bayes-B, respectively (Fig. 1g–i). The negligible effect of heritability in this instance is likely a consequence of the large sample size and low *N*_*e*_ used in the present investigation (Meuwissen et al. 2001).

### Pedigree vs. marker-based models

Although some results were similar, most predictive accuracies and especially predictive abilities for GS models trained on EBVs were still lower than those of ABLUP (Tables 2 and 3), a common issue in forestry (Bartholomé et al. 2016). It is thought that since the Douglas-fir genome is so exceedingly large and complex (Neale et al. 2017), many more markers will be needed in order to track LD between sources of variation and markers (Thistlethwaite et al. 2017). Simulations have yielded evidence that GS prediction accuracy increases markedly with marker density at least up to 8*N*_*e*_/Morgan (Solberg et al. 2008). In this study, we have been moderately successful in capturing both contemporary and historical pedigree information. Although this is somewhat driven by groupings in the validation generation (F_2_) characterized by high and low juvenile height values. Further investigation led to the discovery that the ultimate cause of this clustering was familial grouping. Each cluster contains whole families which can be traced back to distinct parents in the previous generation (F_1_). There are no parents who are represented in both groups. In addition to this, the average heights and EBVs of the parents of the “high” cluster (EBV > 20) were 765.04 cm and 60.70, respectively; and are indeed higher than those parents of the “low” cluster (EBV < 20), 709.88 cm and 0.81, respectively.

Analysis of each cluster provided mixed results. For those scenarios in which the GS models were trained on EBV data (scenarios 1 and 3), the within-cluster results were always lower than the overall predictive accuracy (and ABLUP predictive accuracy) with one exception. Convincing evidence that relationship tracking was the major driving force behind the strong overall correlations. Again within these two scenarios, the training set composition had an effect. Unsurprisingly the analyses with training sets comprised of parents of the cluster in question only, generally gave higher prediction accuracies due to the high relationship between the two sets. Indeed the only scenario in which the GS prediction accuracy surpassed the ABLUP prediction accuracy was for the correlation between HTJ EBVs and HTJ GEBVs with the limited training set EBV > 20^3^ and using Bayes-B as the GS method (0.99 vs. 0.92 for ABLUP). Although in some cases, the effect of training set composition was minimal (scenario 1: EBV < 20 and, Scenario 3: EBV > 20) (Table 2). Taking into account their clustering, predictive abilities for scenarios 1 and 3 fell far short of their ABLUP predictive abilities (Table 3). With the exception of the correlation between HTJ and HTJ GEBVs, which showed moderate success with a value of 0.59. This yet still falling short of the ABLUP value of 0.71. Within-cluster correlations between HTJ and HT35 GEBVs (scenario 3) were all negative, whilst the overall correlations were marginally positive due to the structure of the data as a whole.

Following the removal of family means by deregression (scenarios 2 and 4), the lack of available marker-QTL LD failed to raise the GS prediction accuracies enough to even approximate those of ABLUP. However, Bayes-B notably performed better than both RR-BLUP and GRR in scenario 2 using the correlation between HTJ DEBVs and HTJ GDEBVs of the whole F_2_ validation set as an indication of prediction accuracy (Table 2). A fundamental difference between the GS methods used is that, as opposed to RR-BLUP and GRR, Bayes-B gives different variances to each locus (including zero), therefore it allows for more weight to be put on the causative SNPs. As is evident from the results here, this drives up the prediction accuracy (Meuwissen et al. 2001). No such differences were seen between the GS methods subsequent to cluster analysis in these scenarios. When analyzed to their full extent given the aforementioned data groupings, these deregressed analyses showed a dramatic drop in prediction accuracy. Suggesting the strong correlation was merely driven by tracking family means. Meanwhile, their predictive abilities show some striking trends. In both deregression scenarios (2 and 4) the EBV < 20 cluster has only negative prediction abilities, averaging −0.19 and −0.12 for scenarios 2 and 4, respectively. Conversely, the EBV > 20 cluster has only positive prediction abilities (with one exception), averaging 0.18 and 0.08 for scenarios 2 and 4, respectively. The discrepancy in the direction of the linear relationship casts some doubt on the reliability of the within-cluster results. A likely symptom of the reduced sample size necessitated for these analyses.

Although it is disappointing to not have captured enough LD to raise the prediction accuracy above ABLUP in a diverse validation population, in terms of real-world application there is a positive finding. This use of marker-based selection nevertheless reduces the need for time-intensive practices such as performing specific crosses and building a structured pedigree (El-Kassaby and Lstibůrek 2009; El-Kassaby et al. 2011). Potentially quickening the breeding process, and perhaps off-setting costs involved in genotyping.

### GS across generations

Once GS becomes a viable option for tree breeders it will likely, in most cases, be deployed to select progeny of the training population without the need for explicit crosses and a lengthy testing phase. Bearing this in mind, it is important to validate models across generations rather than cross-validate within the same generation, as many have done before.

GS relies on LD between markers and causal gene variants. LD breaks down after every breeding cycle due to recombination. This is especially pertinent to breeding within the forestry sector, for forest tree species have lower observed levels of LD (Neale and Kremer 2011). Given these circumstances, large SNP sets will be required to provide dense coverage of the genome to find LD between SNPs and QTL (Jaramillo-Correa et al. 2015). In a simulation study, it was shown that higher marker densities allowed prediction accuracies to be maintained over longer generational gaps (Müller et al. 2017b). Thus, without addressing this issue, predictive accuracy is expected to fall dramatically (Habier et al. 2007; Atefi et al. 2016).

There are a reported 54,830 gene models in the Douglas-fir genome (Neale et al. 2017). With our 69,551 SNPs, using the juvenile height of an F_1_ generation as the training set and an F_2_ generation as the validation set, we obtained an overall predictive accuracy of 0.92, 0.91, and 0.91 for RR-BLUP, GRR, and Bayes-B, respectively for juvenile height. This is in line with results from a previous study of GS in Douglas-fir (0.79–0.92) using cross-validation within the same generation (Thistlethwaite et al. 2017).

The results we present here did not show a drop in predictive accuracy as one would expect (with a caveat described later). As has been shown before, prediction accuracy increases when the genetic relationship between training and validation sets is closer (Habier et al., 2007; Lorenz et al. 2012; Sallam et al. 2015). Indeed, it is the case here that our validation set is closely related to the training set by virtue of being their (the training set’s) offspring. Although our results are not as disparate, Bartholomé et al. (2016) observed a similar trend. When progeny validation was used, prediction accuracies of GS methods were higher (0.70 using genomic BLUP (GBLUP), and 0.71 using Bayesian LASSO (B-LASSO)) than when within generation cross-validation was carried out (0.66, for both GBLUP and B-LASSO). With that in mind, there may be some benefit in combining phenotypes across generations which helps to infer the Mendelian sampling term.

However, as is evident from Fig. 1a–c, g–i, two distinct data groups had arisen which had caused the high correlations. These two groups were characterized by (a) those individuals with low juvenile height values (mean = 641.81 cm) and (b) those with high juvenile height values (mean = 696.79 cm). They were later found to be the result of familial clusters, with no overlapping parents from group to group. The estimation of breeding values exacerbated this trend and thus two non-overlapping groups can be seen defined as EBV < 20 (individuals with EBVs less than 20) and EBV > 20 (individuals with EBV over 20).

Given this data structure, further analysis showed that prediction accuracies for scenario 1 EBV < 20 were in fact only moderate, with an average of 0.42 for all GS methods compared to ABLUP (0.92). In the case of EBV > 20 scenario 1 results were close to zero with the exception of EBV > 20^3^. High prediction accuracies, in this case, were driven by close relationships between the training and validation sets and small sample size.

These results might possibly be improved by re-estimation of EBVs using a realized relationship matrix (***G***-matrix) rather than the estimated relationship ***A***-matrix used here (Munoz et al. 2014).

Prediction accuracies for deregressed values reported here, align with those reported in an earlier investigation also carried out on Douglas-fir. Thistlethwaite et al. (2017) describe obtaining prediction accuracies for GS models, trained on DEBVs of height at age 12, that were also approximately 0. These models were cross-validated with individuals from within the same generation. Here, using similar parameters but using a cross-generational validation process, we have obtained similar results with a few exceptions. Once data grouping was taken into account, correlations between HTJ DEBVs and HTJ GDEBVs (scenario 2) were low-moderately negative, −0.12 to −0.02 for EBV < 20 and −0.23 to 0.26 for EBV > 20.

Although a lower predictive accuracy for F_2_ HTJ was obtained when the GS models were trained on F_1_ mature height (age 35) (scenario 3), there was still a significant positive correlation between the two (0.57, 0.56, and 0.58 for RR-BLUP, GRR, and Bayes-B, respectively). These results are lower but similar to findings in Thistlethwaite et al. (2017) where a positive time–time correlation between juvenile (age 12) and mature (age 35) height within the same generation was found (0.71 (SE 0.0004) for both RR-BLUP and GRR). However here again, the groups EBV < 20 and EBV > 20 were the driving force behind these correlations. For scenario 3 EBV < 20 results were likewise reduced, averaging 0.12 for EBV < 20^1^ and EBV < 20^2^, and 0.37 for EBV < 20^3^ across all GS methods. The stronger correlation coming from the increased relationship between the training and validation sets, yet falling quite short compared to ABLUP (0.60). In scenario 3 the EBV > 20 accuracy results show a small-moderate but negative correlation averaging −0.25 across all GS methods. Marker-trait associations vary with tree age, and predictably the ABLUP correlation here was found to be lower than in scenario 1 (0.92) at 0.60. The high-positive EBV > 20^3^ juvenile–juvenile correlation and small-moderate negative EBV > 20 mature-juvenile correlation, may also be symptoms of the effect of tree age on marker-trait associations (Lerceteau et al. 2001) and as seen here, recombination. These results do not provide a sound basis on which to perform selection decisions. However should there be an improvement in GS accuracy with the re-estimation of EBVs, useful information may still be procured from moderate correlations, for input into early selection decisions.

In the final scenario, deregressed breeding values for mature height in the F_1_ generation were used as training data for the GS models. Predictive accuracy fell significantly both for the validation set as a whole (−0.15 to 0.11), and for each of EBV < 20 (−0.08 to 0.07) and EBV > 20 (−0.07 to 0.08).

In the absence of average parental information (i.e., deregressed phenotypes), the ability of the markers to predict phenotypes in the next generation was consistently poor (Table 2). None of the GS analyses in these deregressed scenarios (2 and 4) surpassed or even matched the predictive ability of the ABLUP models, despite having very similar overall prediction accuracies in scenarios 1 and 3 (although this itself can be attributed to the effects of clustering in the validation set). Predictive ability similarly dropped dramatically after deregression and re-adjusting for clustering. The opposing signs of coefficients for the high and low clusters after deregression suggest some unreliability of these values. This trend is reflected in throughout, thus any of these within-cluster GS “accuracies” should be treated with caution.

### Main factors that affect GS: relatedness and LD

Whilst some of our observations during this study align with previously published work, the high predictive accuracies using EBVs as the model input are a result of tracking family means as opposed to true LD being captured. GS BLUP methods are robust in most circumstances and perform well; however, it is now known that these methods primarily capture marker derived relatedness more readily than actual LD (Habier et al. 2007; Zhong et al. 2009). We base this viewpoint on trends seen in Thistlethwaite et al. (2017) whereby removing family derived means from trait breeding values, caused virtually no prediction accuracies of GS methods. Simulation evidence suggests that pedigree-based relationships contribute to predictive accuracy for a few generations, especially given low *N*_*e*_ (Müller et al. 2017b), which the progeny validation set presented here certainly does have. In empirical studies, there is also evidence that the relatedness of the training and validation sets has an impact on the accuracy of GS (Resende et al. 2012a; Beaulieu et al. 2014a; Bartholomé et al. 2016; Märtens et al. 2016; Varshney et al. 2017). With higher relatedness between sets producing more accurate predictions. Our training and validation set are only a generation apart; it is therefore likely that their relatedness is the main force behind the GS prediction accuracies. Even though the alternative, more computationally intense GS method of Bayes-B was employed to help overcome this (Habier et al. 2007), we have not been able to resolve any additional LD using this method. In order to “reveal” the effect of LD on GS predictive accuracy, empirical investigations on multiple generations further apart will have to be conducted using much denser SNP genotyping.

Apropos of marker density, we carried out analyses concerning the effect of marker density on the prediction accuracy of GS. We carried out a tenfold cross-validation on HT35 EBVs within the genotyped parental (F_1_) generation samples using RR-BLUP. Randomly selected marker sets were chosen that had progressive totals from 200 to 50,000 SNPs. These sets were tested and replicated ten times, with random SNP sampling for each repetition. Our investigations led us to the conclusion that an increase in marker density leads to an increase in GS prediction accuracy. A caveat being that the magnitude of prediction accuracy gains falls to a plateau as the number of makers approaches approximately 15,000 SNPs. This also happens to be the point at which the prediction accuracy is similar to the ABLUP prediction accuracy for HT35 EBVs for these samples. When the same method was employed on deregressed HT35 EBVs, the prediction accuracies fell dramatically for all SNP set totals. We believe that relatedness is the driving force behind these additional GS predictive accuracy results for the HT35 EBVs for a couple of reasons. Firstly, the variance within each SNP set total was modest even though the SNPs were randomly selected. Secondly, the prediction accuracies fell dramatically when the EBVs were deregressed to remove the parental average effects, for the purposes of extricating LD from pedigree. Given this insight, in order to capture short-range marker-QTL LD in conifer species, we recommend the use of deregression to remove parental average effects and high-density marker sets.

The effect of capturing marker derived relatedness may lead to sibling coselection due to an increased correlation between EBVs within families (Wray and Thompson 1990). In the long-run, this will lead to a reduction in genetic variation in subsequent generations, and a loss of potential genetic gain (Hallander 2009). A reduction in Mendelian segregation variance due to inbreeding (sibling coselection) will eventually lead to a decrease in the Mendelian sampling term of each individual, and population-wide additive variance. Presenting a problem for breeders in that the expectation of the rate of genetic gain in a population is proportional to the Mendelian sampling term of selection targets (Woolliams and Thompson 1994; Avendaño et al. 2004).

Breeding programs often implement selection methods that optimize the selection differential whilst constrained by a limit on increasing coancestry. Hallander and Waldmann (2009) tested such methods on a diallel progeny trial of Scots pine (*Pinus sylvestris* L.) investigating height and stem diameter and found optimum contribution (OC) dynamic selection resulted in the highest genetic gain over other methods (standard restricted selection). One of the main drivers for the success is that the selective advantage in the OC method is the Mendelian sapling term. The OC method maximizes the selection differential between families by using the best estimates of the Mendelian sampling term for each tree when calculating the mating contributions (Avendaño et al. 2004). Selection methods must be considered when using GS results as a basis for selection, to avoid inbreeding and to maintain Mendelian segregation variance.

## Conclusions

Our cross-generational GS validation of juvenile height in Douglas-fir provided results that almost matched the ABLUP predictive accuracy. However, we believe that the predictive accuracy is driven by the relatedness between the training and validation sets, and even more so in capturing among-family effects. Whilst this relationship is exploited for selection in current breeding programs, the ultimate aim of using GS should be to capture true LD across populations and traits to uncover presently unknown variation, and possibly unknown traits (Beaulieu et al. 2014a; Grattapaglia 2017). Hence, we degregressed EBVs to tease apart LD from familial relationships, which may subsequently be preferential only in advanced breeding programs (Grattapaglia 2017). While the number of SNPs we have derived from sequence capture represents the highest in forest trees GS studies (Grattapaglia 2017), we have yet to observe enough marker-QTL LD in our GS methods. Many more markers may be required for this to be resolved since LD in Douglas-fir survives only over short distances as in other conifer species (Neale and Savolainen 2004). In addition, multi-generational information may be required in order to evaluate LD in this species. This must be explored further before any such incorporation into applied selective breeding programs is undertaken.

### Data availability

The data sets supporting the results of this article are available from the Dryad Digital Repository 10.5061/dryad.8n2d374.
